# Glass Ionomer Cements with Halloysite-CHX: Physicochemical Properties, Antimicrobial Activity, Cell Viability

**DOI:** 10.1590/0103-644020256350

**Published:** 2025-07-11

**Authors:** Fabiola Rodrigues Sampaio Nunes, Thaís Bezerra da Maceno Oliveira, Shirley Maria de Nazaré Rocha Cardoso, Rayssa Ferreira Cavaleiro de Macêdo, Ana Paula Silva de Azevedo dos Santos, Paulo Vitor Campos Ferreira, José Bauer

**Affiliations:** 1 Programa de Pós- Graduação em Odontologia, Laboratório de Biomateriais do Maranhão (BIOMMA), Universidade Federal do Maranhão, São Luís, Brasil; 2 Laboratory for Applied Cancer Immunology, Biological and Health Sciences Center, Federal University of Maranhão(UFMA), Zip Code 65080-805 São Luís, MA, Brazil

**Keywords:** Glass ionomer cement, halloysite nanotubes, chlorhexidine, antimicrobial activity, mechanical properties

## Abstract

This study evaluated the physicochemical, cell viability, and antimicrobial properties of conventional glass ionomer cement (GIC) modified by the incorporation of halloysite nanotubes (HNT) doped with chlorhexidine (CHX). HNT-CHX was added to GIC at concentrations of 2.5% and 5% by weight, forming three experimental groups: a control group (GIC only), a group with 2.5% HNT-CHX, and another with 5% HNT-CHX. Hourglass-shaped specimens (n=10) were used to measure cohesive strength and elastic modulus, while fractured specimens were subjected to microhardness testing (n = 5). To assess the alkalinizing activity (pH) and the release of F-, Ca^+2^, and PO4^-3^ ions (n = 3), discs were prepared for CHX release analysis by UV-vis after 24 hours. Antimicrobial activity was tested against *S. mutans* biofilm (CFU/ml), and material cell viability was determined using the MTT assay. Results showed that the 5% HNT-CHX group presented the lowest cohesive strength, while the 2.5% and 5% HNT-CHX groups displayed modulus values similar to the control and did not affect microhardness. All groups exhibited an acidic pH with the control group releasing higher levels of F^-^, Ca^+2^, and PO_4_^-3^ ions. The HNT-CHX 2.5% and HNT-CHX 5% groups reduced fluoride ion release compared to the control (GIC). The 5% HNT-CHX group showed the highest levels of free CHX and demonstrated bactericidal activity against *S. mutans* in CFU analysis. None of the materials presented cytotoxicity. In conclusion, incorporating 2.5% and 5% HNT-CHX affected the mechanical properties and ion release of the GIC. HNT-CHX 5% group exhibited antimicrobial against *S. mutans*.

## Resumo

Embora amplamente utilizados na odontologia, os Cimentos de Ionômero de Vidro (CIVs) possuem baixo desempenho mecânico, limitando seu uso em áreas de alto estresse. Alterações em sua composição têm sido propostas para melhorar essas propriedades. O objetivo desse estudo foi avaliar as propriedades mecânicas, físico-químicas, viabilidade celular e atividade antimicrobiana de CIV convencional modificado pela adição de nanotubos de haloisita (HNT) carregados com clorexidina (CHX). Foram utilizadas concentrações de 2,5% e 5% de HNT-CHX, incorporadas ao pó do CIV convencional (Bioglass R). Os grupos experimentais incluíram um controle (CIV), HNT-CHX 2,5% e HNT-CHX 5%. As propriedades mecânicas foram avaliadas através de testes de resistência coesiva, módulo de elasticidade e microdureza Knoop. Testes físico-químicos incluíram medição do pH e liberação de íons (F^-^, Ca^+2^, PO_4_-^3^), enquanto a liberação de CHX foi medida por espectroscopia UV-vis após 24 horas. A atividade antimicrobiana foi avaliada contra biofilme de Streptococcus mutans, e a viabilidade celular foi testada pelo ensaio MTT. Os resultados mostraram que o grupo CIV/HNT-CHX 5% apresentou menor resistência coesiva, mas sem diferenças significativas nos valores de módulo de elasticidade comparado ao controle. Não houve alteração na microdureza entre os grupos. Todos os grupos apresentaram pH ácido, com o controle liberando mais íons F-, Ca+2 e PO4-3 do que os grupos com HNT-CHX. O grupo CIV/HNT-CHX 5% mostrou maior liberação de CHX livre após 24 horas, porém nenhum dos grupos apresentou atividade antimicrobiana significativa contra S. mutans. Não foi observada citotoxicidade em nenhum dos materiais testados.

**Figure:**
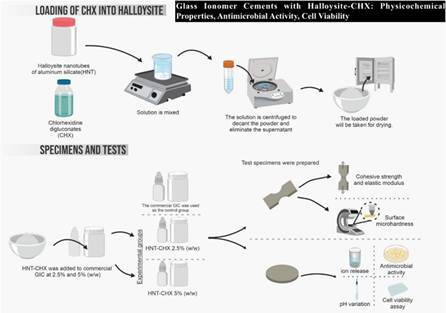

## Introduction

Glass ionomer cements (GIC) are well-established materials in dentistry with significant applications in pediatric clinics due to their ability to chemically adhere to dental structures and, most importantly, their release of fluoride ions ^1^ ^,^ ^2^ ^,^ ^3^. The availability of these ions is associated with the formation of fluoride salts, a process involving an acid-base reaction structured by covalent bonds between the molecules of polycarboxylic acids and the ions leached from the fluoro aluminosilicate particles ^4^. In addition to functioning as reinforcement particles, unreacted particles also serve as sources of F^-^ ions throughout the material degradation process ^5^.

Considering the important role of fluorides in preventing carious lesions, applying GIC is closely associated with minimally invasive techniques, such as Atraumatic Restorative Treatment (ART) ^2^. Some authors correlate this release of F^-^ ions with the inhibition of the enzyme enolase in bacteria of the cariogenic biofilm, which would affect the metabolism of sugars and ATP synthesis, leading to the death of these microorganisms ^6^ ^,^ ^7^ ^,^ ^8^. However, there is no strong evidence in the literature to support this antimicrobial action of GIC.

Despite their prominent use in pediatric clinics, these materials also have other clinical applications, such as lining, bracket bonding, and sealing pits and fissures ^4^. However, their use in restorative procedures in areas of high mechanical stress is limited due to their low mechanical performance, notably low flexural strength, cohesiveness, and wear resistance ^9^ ^,^ ^10^ ^,^ ^11^.

The addition of bactericidal and/or bacteriostatic agents has been the subject of studies to enhance the use of these materials in minimally invasive techniques ^10^ ^,^ ^12^. Among the main antimicrobial agents, chlorhexidine (CHX) is a potent inhibitor of enzymatic and bacterial activity, and it has varied applications as it is a broad-spectrum cationic bactericide ^12^. In restorative treatments, 2% CHX can be used as a re-wetting agent for the collagen layer ^13^. However, besides adding an extra step to the restorative protocol, this application of CHX has controversial evidence of long-term efficacy ^14^ ^,^ ^15^. Takahashi et al. (2006) ^12^ reported that chlorhexidine (CHX) can also affect the adhesion and setting time of GICs when added at high concentrations due to the presence of CHX salts and the cationic properties of CHX; they also suggested the incorporation of 1% chlorhexidine diacetate as the optimal concentration to achieve satisfactory physical, antimicrobial, and bonding properties.

Drug delivery systems have been extensively studied as an alternative for incorporating and optimizing these compounds into dental material matrices ^16^. Among the various available systems, halloysite nanotubes (HNT) have been recently employed in the development of dentin adhesive systems loaded with chlorhexidine ^17^. The use of these nanostructures did not alter the degree of conversion and viscosity of these materials; moreover, they imparted significant antimicrobial potential to the material. Halloysite nanotubes (HNT) are naturally occurring aluminosilicate clay minerals that are biologically safe and capable of retaining and releasing molecules in a controlled manner through surface desorption and the diameter of the tube ends ^18^ ^,^ ^19^. Halloysite nanotubes show the chemical formula Al₂Si₂O₅(OH)₄, capable of encapsulating, storing, and transporting various drugs ^20^. These nanostructures exhibit biocompatibility, hydrophilicity, and high mechanical strength, and such characteristics allow their application in different matrices and perform dual functions, such as mechanical reinforcement and drug delivery ^21^.

Based on the presented ideas, we formulated the null hypothesis that the incorporation of halloysite nanotubes loaded with chlorhexidine into commercial glass ionomer cement does not affect the material's mechanical strength or cause significant changes in its physicochemical properties. To address these questions, the present study evaluated commercial glass ionomer cement's mechanical, physicochemical, and antimicrobial properties after incorporating halloysite nanotubes loaded with chlorhexidine.

### Materials and methods

### Loading of Halloysite Nanotubes with Chlorhexidine (HNT-CHX)

A 20% aqueous solution of chlorhexidine was used to load halloysite nanotubes of aluminum silicate (Halloysite Nanoclay, Sigma Aldrich, St. Louis, MO, USA). The HNT powder was mixed with the CHX solution (at a ratio of 1.25 g of HNT powder per 5 mL of CHX solution). The HNT-CHX mixture was stirred for 1 hour on a magnetic stirrer (Heidolph, MR Hei-Tec, Germany) at 37°C and then centrifuged (Kasvi, K14-4000, China) at 4000 rpm for 5 minutes. The supernatant was removed, and the precipitate was washed and dried in an oven at 37°C for 7 days ^15^.

### Sample Preparation

Control specimens (GIC) were prepared according to the manufacturer's instructions for Bioglass R (Biodinâmica, Paraná, Brazil). Experimental GICs were prepared by incorporating two concentrations (2.5% and 5% by weight) of nanotubes loaded with a 20% aqueous chlorhexidine solution (HNT-CHX) into Bioglass R powder (Biodinâmica) using a precision balance (Shimadzu, AUW220D, Japan), and vortexed (KASVI, K45-2820, Jiangsu, China) for 1 minute for powder homogenization ^22^.

GIC handling involved mixing on paper blocks using a plastic spatula for 30 seconds until powder-liquid homogenization. The material was placed into silicone molds with specific dimensions for each test, the excess was removed, and a polyester strip and glass lamina were positioned on the surface. After allowing 10 minutes for the initial setting, specimens were kept in moist gauze for 1 hour to prevent material syneresis.

### Mechanical Properties

### Cohesive strength and modulus of elasticity

Hourglass-shaped specimens (10 mm length, 2 mm width, and 1 mm thickness) with a cross-sectional area of 1 mm² were prepared for each group (n = 10). The specimens were then stored in an oven at 37 °C with humidity control for 24 hours and subjected to cohesive strength tests using a universal testing machine (Instron 3342, Massachusetts, USA) at a 1 mm/min rate until failure ^22^. Load values in Newtons (N) were converted to Megapascals (MPa). Elastic modulus data were captured from the stress-strain curve's linear portion using BlueHill software.

### Knoop microhardness (KHN)

Fractured specimens from the previous test (half-hourglass) were selected, embedded in acrylic resin, and polished using an Aropol E polishing machine (Arotec S.A Indústria e Comércio, São Paulo, Brazil). Subsequently, an HMV-G20 microhardness tester (Shimadzu -Future- Tech Corporation, Tokyo, Japan) was used under the following parameters: 40X lens, with a 0.01 KgF load applied for 10 seconds, to make four different indentations per specimen and analyze their surface microhardness ^22^.

### Physicochemical Properties

### Alkalinization activity (pH)

Disk-shaped specimens (10 mm diameter × 2 mm thickness) were prepared (n = 3). After spatulation, specimens were immersed in 8 ml of deionized/distilled water at an initial pH of 7 (pH_i_ = 7) for 28 days. pH readings were taken at 15 minutes, 30 minutes, 60 minutes, and 28 days using a pH digital analyzer with coupled electrode (Quimis, Diadema, SP, Brazil). After the final reading, discs were removed, and the solution was analyzed for fluoride ions (F^−^), PO_4_^-3^, and calcium ions (Ca^2+^) ^23^.

### Fluoride ions release (F- )

Fluoride ion release (F^−^) was measured using an ion-selective electrode for F^−^ (Quimis, Model Q400ISE, Diadema, SP, Brazil), coupled with a pH and fluoride digital analyzer (Alyser Fluoreto 18AF - 001). The device was calibrated with standard solutions at F^−^ concentrations of 6.0, 12.0, 24.0, 40.0, 48.0, and 96.0 ppm, post-buffered with TISAB II (Total Ionic Strength Adjustment Buffer) at a 1 mL: 1 mL ratio. Readings were taken in triplicate.

### Calcium (Ca2+) and phosphate (PO4 3- ) ions release

Calcium ion (Ca^+2^) quantification used an arsenazo colorimetric reagent (Calcium Arsenazo III, Doles, lot # 13051, Goiânia, GO, Brazil), and phosphate used a colorimetric reagent (Phosphate, Doles, lot # 14041, Goiânia, GO, Brazil). Ca^+2^ and PO_4_^-3^ ion amounts were determined from three repeated readings per sample using a spectrophotometer (ELX800, Biotek, Winooski, VT, USA), with an arithmetic mean of absorbance at 650 nm ^24^.

### Chlorhexidine release

For chlorhexidine release analysis, disk-shaped specimens (2 mm × 10 mm) from each study group were made in triplicate and stored in ultrapure water for 24 hours (ratio of 1 specimen to 2 mL water). Afterward, liquid portions were analyzed via ultraviolet-visible spectroscopy (UV-vis) (Spectrophotometer Spectramax 190, Molecular Devices, Sunnyvale, CA, USA). Results were obtained through absorbance, converted to mg/mL using a calibration curve equation ^25^.

### Antimicrobial Activity

Three samples from each group were prepared from matrices (10 mm × 2 mm) fixed to the lid of a 24-well plate to assess antimicrobial activity. Frozen aliquots of *Streptococcus Mutans* (UA159) were deposited on BHI agar plates (BHI; Sigma-Aldrich, St Louis, MO, USA) and incubated for 48 hours at 37 °C. Colony-forming units (CFU) were collected and transferred to tubes containing BHI broth supplemented with 1% sucrose and grown until the late experimental phase. To form a microbial inoculum, suspensions were adjusted using the 0.5 McFarland scale standard solution, resulting in a suspension with an approximate concentration of 108 CFU/mL.

Aliquots of BHI (1 mL ) supplemented with 1% sucrose were added to each well of a sterile 24-well plate, followed by 100 µl of adjusted microbial solution. The lid containing sterilized samples was placed on the plate. All samples were immersed in broth and inoculum suspension. The plate was incubated for 24 hours at 37°C. After 24 hours, 100 µl aliquot from each well was transferred to tubes containing 1 ml sterile saline solution and vigorously vortexed. Serial dilutions of aliquots were performed to reach a dilution of 10^-8^, and 20 µl drops of each dilution were inoculated onto BHI agar (BD, Sparks, USA) to determine the number of colony-forming units. Plates were incubated for 48 hours at 37°C, 10% CO_2_. After 48 hours, colony-forming units were counted using a stereomicroscope. Results were expressed in log10 CFU/mL. Chlorhexidine solution at a concentration of 20% was used as a positive control.

### Cell Viability - MTT Assay

For cytotoxicity testing, an immortalized human fibroblast cell line (GM07492A) was cultured in Dulbecco's Modified Eagle Medium (DMEM) supplemented with 10% fetal bovine serum (FBS) and 1% penicillin/streptomycin and incubated (37 °C, 5% CO_2_, and 95% air). Cells were adjusted to a concentration of 10^4^ cells/mL and cultured in a 96-well plate (n=3/group) for 24 hours. Simultaneously, specimens were prepared, sterilized with ultraviolet light (30 minutes), and immersed in 4 mL of DMEM for 24 hours. Subsequently, cells were treated with DMEM, which was used to immerse experimental adhesive samples and analyzed after 24 and 48 hours of contact (37 °C, 5% CO_2_, and 95% air). Cell viability was determined by colorimetric MTT reduction assay at a wavelength of 570 nm.

### Statistical analysis

Statistical analysis was performed using SigmaPlot software (Systat Software Inc., San Jose, California, USA). Data normality was tested with the Shapiro-Wilk test (P>0.05) and equality of variances with the Brown-Forsythe test (P > 0.05). Antimicrobial activity (CFU) data violated normality (p < 0.05) and were transformed into Log10 (CFU/mL), analyzed by the Kruskal-Wallis and Student-Newman-Keuls tests for the contrast between the means. Cohesive strength, elastic modulus, Knoop microhardness (KHN), fluoride ion release (F⁻), calcium ion release (Ca²⁺), phosphate ion release (PO₄³⁻), cell viability, and chlorhexidine release were evaluated using one-way ANOVA followed by Holm-Sidak post hoc tests (α = 0.05). The alkalization activity (pH) and CHX release were expressed as descriptive values.

## Results

### Mechanical Properties

Table 1 presents the mean values and standard deviations of cohesive strength (MPa), elastic modulus (GPa), and Knoop microhardness (KHN) for the different tested groups. The GIC/HNT-CHX 5% group showed the lowest cohesive strength values (p < 0.05), while the GIC/HNT-CHX 2.5% group exhibited values similar to the control group (p > 0.05). Results for elastic modulus indicated that both GIC/HNT-CHX 2.5% and 5% groups had comparable values to the control group (p < 0.05). The addition of different concentrations of HNT-CHX into the GIC matrix did not affect the material's surface microhardness in any of the tested groups (p > 0.05).

**Table 1:** Means and standard deviations of the tested groups' cohesive strength, elastic modulus, and Knoop microhardness values.

Grupos	Cohesive Strength (MPa)	Modulus (GPa)	Microhardness (KHN)
GIC (Control)	7.5 ± 2.0 (a)	1.1 ± 0.3 (a)	23.4 ± 7.3 (a)
GIC-CHX 2.5%	7.1 ± 2.4 (a)	0.7 ± 0.2 (b)	25.6 ± 5.9 (a)
GIC-CHX 5%	4.5 ± 1.6 (b)	0.5 ± 0.3 (b)	27.5 ± 7.8 (a)

*Different letters in the same column indicate statistically significant differences between groups (p < 0.05).

### Physicochemical Properties

All groups exhibited acidic pH values below seven at the evaluated reading intervals (15, 30, 60 minutes, and 28 days).

Table 2 presents the mean values and standard deviations of fluoride, calcium, and phosphate ion release obtained for the tested groups. The control group showed higher F^-^ release compared to the groups containing HNT-CHX at 2.5% and 5% (p > 0.05) after 28 days of storage. The groups containing HNT-CHX released fewer Ca^+2^ and PO_4_^3-^ ions compared to the control group (p > 0.05).

**Table 2:** Results of fluoride, calcium, and phosphate ions released from the tested groups.*

Groups	Fluoride	Calcium	Phosphate
GIC (control)	77.2 ± 12.8 (a)	1.2 ± 0.4 (a)	2.9 ± 0.5 (a)
HNT-CHX 2.5%	50.1 ± 3.7 (b)	0.5 ± 0.07 (b)	0.6 ± 0.4 (b)
HNT-CHX 5%	52.3 ± 5.8 (b)	0.5 ± 0.1 (b)	0.5 ± 0.5 (b)

*Different letters in the same column indicate statistically significant differences between groups (p < 0.05).

### Chlorhexidine release

The HNT-CHX 5% group showed the highest values of free CHX after 24 hours of analysis (0.13 mg/ml), while the HNT-CHX 2.5% group obtained 0.10 mg/ml. The control group did not release chlorhexidine.

### Antibacterial Activity

**Figure 1:**
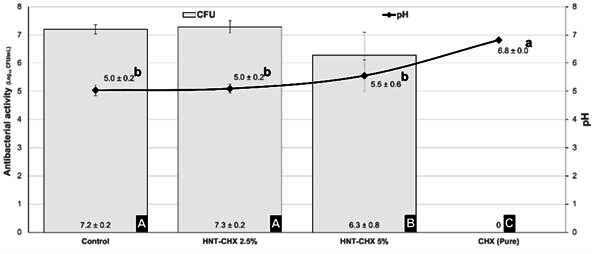
Mean and standard deviation of CFU values of antimicrobial activity and pH of the bacterial medium from the tested groups*.

Figure 1 shows the tested experimental groups' mean values and standard deviations of antimicrobial activity. The groups GIC (control) and HNT-CHX 2.5% showed similar values of antimicrobial activity (p > 0.05). The group HNT-CHX 5% showed antimicrobial activity superior to the GIC control and HNT-CHX 2.5% groups (p<0.05). Pure chlorhexidine exhibits a high antimicrobial effect against S. mutans (p < 0.05). The pH of the bacterial medium was acidic for the GIC (control), HNT-CHX 2.5%, and HNT-CHX 5% groups, indicating a medium rich in bacteria-producing acidic products. Pure chlorhexidine exhibited a pH very close to neutrality (pH=7) and statistically higher than the other experimental groups (p < 0.05).

### Cell viability - MTT assay

**Figure 2:**
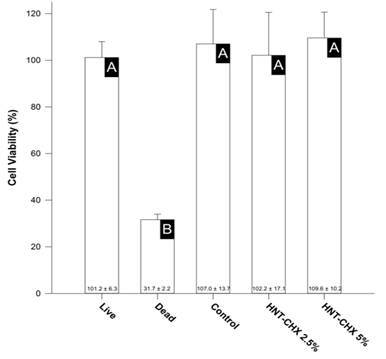
Mean and standard deviation of cell viability (%) of the groups tested.

Samples from all experimental groups showed similar biocompatibility values, being noncytotoxic for cells (p > 0.05) (Figure 2).

*Different uppercase letters indicate statistically significant differences between the groups in antibacterial activity (p < 0.05).

** Different lowercase letters indicate statistically significant differences between the groups in the pH of the bacterial medium (p < 0.05).

* Different letters indicate statistically significant differences between groups (p < 0.05).

## Discussion

Due to some limitations of this material, the properties of glass ionomer cement have been constantly investigated through modifications in its composition. These modifications primarily aim at mechanical reinforcement and the attribution of antimicrobial activity ^9^ ^,^ ^10^ ^,^ ^11^.

In this study, halloysite nanotubes loaded with 20% chlorhexidine digluconate were incorporated into a commercial glass ionomer cement powder. The effects of this modification on the matrix formation kinetics, mechanical, and physicochemical properties of the glass ionomer cement were evaluated, as well as the presence of antimicrobial activity. The null hypothesis was rejected, as the incorporation of chlorhexidine-loaded halloysite nanotubes into commercial glass ionomer cement negatively affected the ionic release of the experimental GICs and reduced the cohesive strength of the HNT-CHX 5% group.

In dentistry, these nanostructures have been applied in the development of adhesive systems to date ^18^. Bottino et al. (2013) incorporated different concentrations of HNT into conventional adhesive systems and demonstrated that, at concentrations above 20%, the degree of conversion was reduced. Additionally, Feitosa et al. (2018) incorporated HNT particles loaded with chlorhexidine into commercial adhesive systems and demonstrated sufficient CHX release to inhibit bacterial growth without compromising the degree of conversion and mechanical properties.

Unlike dimethacrylate polymers, such as adhesive systems, glass ionomer cement have a sensitive gelation process ^8^. The formation of these materials involves an acid-base reaction between polyalkenoic acid copolymers and fluoroaluminosilicate particles, and this process is intrinsically related to the powder-liquid ratio of the material ^1^ ^,^ ^2^ ^,^ ^3^. Thus, the insertion of external agents into this reaction can interfere with the matrix formation kinetics and influence the material's properties, as pointed out by the results of the cohesive strength test conducted in this study.

The cohesion values showed a reduction for the HNT-CHX 5% experimental group. On the other hand, when at a lower concentration, HNT-CHX 2.5%, the values were similar to the control group. However, the modulus of elasticity was lower than that of the control group in both groups containing HNT-CHX. Similar results were observed in a study that used mesoporous silica nanoparticles as CHX carriers inserted into conventional glass ionomer cement ^21^.

Still, as a consequence of the disturbance to the reaction kinetics, ionic dissolution was also altered in this study. The ability to release F^-^ ions is an important property of GICs and is directly related to the anticariogenic effect exerted by these materials ^20^ ^,^ ^21^. Fluoride release is influenced by intrinsic factors, such as matrix composition and fluoride content, and extrinsic factors, such as medium pH, powder/liquid ratio, mixing, setting time, and exposed surface area ^7^. The release data from this experiment indicate a reduction of approximately 35% in F^-^ ions available in the solution for both HNT-CHX groups compared to the control group. An even more pronounced reduction was observed for Ca^+2^ and PO_4_^-3^ ions. These findings reinforce the interference of HNT insertion in the formation process of the ionomeric matrix and are consistent with other studies that added different compounds to GIC ^13^ ^,^ ^15^.

In contrast to the negative impact caused by the incorporation of HNT-CHX on mechanical properties and ion release, the addition of HNT-CHX in the experimental group HNT-CHX 5% exhibited antimicrobial action, differing from the other groups. This result is directly related to the higher chlorhexidine release capacity. The HNT-CHX 2.5% group could also release CHX; however, the amount was insufficient to reduce bacterial colonies during the evaluated period. Similar results were observed in the study by Yan et al. (2017), which utilized mesoporous silica particles as CHX carriers. The incorporation of CHX-loaded nanoparticles also reduced mechanical properties; however, antimicrobial activity was observed in all groups containing MSN-CHX ^21^.

This study shows that the incorporation of HNT-CHX affects the mechanical properties of the material, as well as interferes with the release of F^-^, an important ion that plays a role in the remineralization of dental tissues. The addition of elements that do not participate in the setting reaction of the GIC and still interfere with this kinetics is always detrimental to the already low mechanical properties of the GIC. It is a consensus in the literature that this alteration in the GIC, even leading to gains in its antibacterial activity, can cause damage to other properties.

On the other hand, further studies should continue to seek alternatives to enhance the antibacterial activity of glass ionomer cement (GIC). Utilizing the GIC particle itself as a drug carrier may serve as an excellent alternative to avoid compromising the material's other properties by adding elements that do not participate in the reaction and act as points of weakness in the GIC. The incorporation of HNT-CHX conferred antimicrobial activity to the GIC at a concentration of 5% without exhibiting cytotoxicity. However, it is noted that this addition impairs certain mechanical properties and the release of essential ions responsible for the remineralization of dental substrates.
